# Polycystic Ovary Syndrome: Aggressive or Protective Factor for the Retina? Evaluation of Macular Thickness and Retinal Nerve Fiber Layers Using High-Definition Optical Coherence Tomography

**DOI:** 10.1155/2015/193078

**Published:** 2015-02-11

**Authors:** José Edvan de Souza-Júnior, Carlos Alexandre de Amorim Garcia, Elvira Maria Mafaldo Soares, Técia Maria Oliveira Maranhão, Telma Maria Araújo Moura Lemos, George Dantas Azevedo

**Affiliations:** ^1^Post-Graduate Program in Health Sciences, Federal University of Rio Grande do Norte, Natal, RN, Brazil; ^2^Department of Biomedical Sciences, State University of Rio Grande do Norte, Mossoró, RN, Brazil; ^3^Department of Biomedical Sciences, Faculty of Health Sciences, Federal University of Rio Grande do Norte, Central Campus, St. Atirador Miguel Antônio da Silva Neto, s/n, Aeroporto, 59607-360 Mossóro, RN, Brazil; ^4^Department of Surgery, Onofre Lopes University Hospital, Federal University of Rio Grande do Norte, Natal, RN, Brazil; ^5^Department of Obstetrics and Gynecology, Federal University of Rio Grande do Norte, Natal, RN, Brazil; ^6^Department of Clinical and Toxicological Analyses, Federal University of Rio Grande do Norte, Natal, RN, Brazil; ^7^Department of Morphology, Federal University of Rio Grande do Norte, Natal, RN, Brazil

## Abstract

*Objective*. To compare macular thickness (MT) and retinal nerve fiber layers (RNFL) between women with polycystic ovary syndrome (PCOS) and healthy women. *Materials and Methods*. The study included 45 women with PCOS and 47 ovulatory women undergoing clinical-gynecological and ophthalmic evaluations, including measurement of MT, RNFL, and optic disc parameters using optical coherence tomography. *Results*. The superior RNFL around the optic nerve was significantly thicker in PCOS than in healthy volunteers (*P* = 0.036). After stratification according to insulin resistance, the temporal inner macula (TIM), the inferior inner macula (IIM), the nasal inner macula (NIM), and the nasal outer macula (NOM) were significantly thicker in PCOS group than in control group (*P* < 0.05). Both the presence of obesity associated with insulin resistance (*P* = 0.037) and glucose intolerance (*P* = 0.001) were associated with significant increase in the PC1 mean score, relative to MT. A significant increase in the PC2 mean score occurred when considering the presence of metabolic syndrome (*P* < 0.0001). There was a significant interaction between obesity and inflammation in a decreasing mean PC2 score relative to macular RNFL thickness (*P* = 0.034). *Conclusion*. Decreased macular RNFL thickness and increased total MT are associated with metabolic abnormalities, while increased RNFL thickness around the optic nerve is associated with hormonal changes inherent in PCOS.

## 1. Introduction

Polycystic ovary syndrome (PCOS) is a complex and heterogeneous endocrine-gynecological disorder that affects approximately 5–10% of women of reproductive age and is the most common endocrine disorder in premenopausal American women [1–3]. It is characterized by the variable presence of menstrual irregularity, hyperandrogenism, and microcystic ovaries [4]. It may be associated with metabolic syndrome [5], insulin resistance [2, 6–9], glucose intolerance, type 2 diabetes [10], cardiovascular disease, obesity, and dyslipidemia [3, 6, 11–13].

From 1964 to the present day, there has been interest in studying the relationship between PCOS and eye health [14–20]. Given the frequent association of PCOS with metabolic syndrome (MS) and changes in glucose metabolism, this study seeks to investigate the presence of early retinal changes in women with PCOS as a strategy to prevent ocular involvement. Furthermore, the presence of sex steroid hormone receptors in various ocular tissues, especially the retina and choroid, leads us to believe that hormonal changes such as hyperandrogenism may also cause changes in the posterior segment of the eye, increasing the potential impact on visual function in these patients [21].

High-definition spectral-domain optical coherence tomography (HD-OCT) is a noninvasive test that enables early detection of subclinical retinal changes to the microvascular level and that provides detailed information on the retinal microstructure with high resolution, accuracy, and reproducibility. The detection of such changes may offer new perspectives for early diagnosis and better understanding of the pathophysiological mechanisms of the posterior segment eye diseases [19, 20, 22–24]. Studies with diabetic patients have shown an increase in total macular retinal thickness and thinning of the retinal nerve fiber layer (RNFL) in the macular region, regardless of the presence of diabetic retinopathy (DR), when compared with healthy volunteers [22, 25]. Initial studies in patients with PCOS have demonstrated increased RNFL thickness around the optic nerve compared with healthy women and its association with the presence of hyperandrogenism [19]. Given the lack of conclusive data on retinal changes in patients with PCOS, this study aims to evaluate total macular thickness and RNFL in the macula and around the optic disc in women with PCOS using HD-OCT and to compare the results with those of healthy ovulatory patients stratified by the presence of metabolic syndrome, inflammation, obesity, and glucose intolerance/insulin resistance.

## 2. Materials and Methods

This cross-sectional study involved women aged 18 to 34 years who were divided into two groups: the PCOS group composed of 45 volunteers with a diagnosis of PCOS and a control group consisting of 47 healthy ovulatory volunteers. Participants were recruited from the outpatient clinics of Januário Cicco Maternity School (Maternidade Escola Januário Cicco), and they were examined in the Ophthalmologic Clinic of the Onofre Lopes University Hospital (Hospital Universitário Onofre Lopes), Federal University of Rio Grande do Norte (UFRN), between May 2012 and March 2013. The study was approved by the Institutional Ethics Committee (Protocol 586/11), and volunteers signed informed consent forms.

Inclusion criteria for the PCOS group were based on diagnostic criteria established by the Rotterdam Consensus (*The Rotterdam ESHRE/ASRM-Sponsored PCOS consensus workshop *group, 2004), according to which the syndrome should be diagnosed when two of the following parameters are present: menstrual disorder, such as oligomenorrhea and/or amenorrhea; clinical and/or laboratory findings of hyperandrogenism; and ultrasound evidence of polycystic ovaries. The control group consisted of volunteers who had regular menstrual cycles, absence of clinical and/or laboratory signs of hyperandrogenism, and ovulation confirmed by progesterone measurements taken between the 21st and 22nd days of the menstrual cycle.

Exclusion criteria included women with other endocrine disorders, renal or liver failure; chronic users of drugs that might interfere with the metabolism of carbohydrates, lipids and with renal function (such as diuretics, antihypertensives, antilipemic agents, and corticosteroids); impairment of hepatic and/or renal function; smoking; alcohol consumption; or illicit drug use. Women who had any eye disease or who had undergone any ocular surgery were also excluded.

To form the groups, all volunteers were submitted to a rigorous selection procedure that consisted of a general clinical and gynecological examination, evaluation of anthropometric data, transvaginal ultrasonography, evaluation of laboratory parameters (biochemical, hormonal, and inflammatory markers), and a complete eye examination. Gynecological and obstetric histories were recorded, including the occurrence of pregnancies ending in childbirth or miscarriage in the preceding 3 months. Personal history of acute cardiocirculatory disease in the past three months, aspirin and anticoagulant use over the past 15 days, and use of corticosteroids in the last 60 days were also investigated. Lifestyle habits, current and previous histories of other diseases, and family histories of diabetes mellitus, cardiovascular disease, dyslipidemia, and obesity were also assessed. Blood pressure, weight, height, and waist and hip circumference measurements were taken.

The laboratory evaluation consisted of a complete blood count, routine urinalysis, and a 75 g oral glucose tolerance test. Additionally, urea, creatinine, triglycerides, total cholesterol and fractions, ALT (alanine aminotransferase), AST (aspartate aminotransferase), gamma-GT (gamma-glutamyl transpeptidase), SHBG (sex hormone-binding globulin) TSH (thyroid-stimulating hormone), FSH (follicle-stimulating hormone), LH (luteinizing hormone), total testosterone, dehydroepiandrosterone sulfate, androstenedione, insulin, C-reactive protein, TNF-*α* (tumor necrosis factor *α*), and IL-6 (interleukin-6) levels were measured. Measurements of plasma glucose were performed using the glucose oxidase enzymatic method. Levels of total cholesterol, high-density lipoprotein- (HDL-) cholesterol, triglycerides, and other biochemical parameters were measured with colorimetric/enzymatic methods by using commercial kits (BioSystems, Barcelona, Spain). Levels of hormonal and inflammatory parameters were determined by using commercially available diagnostic kits in the IMMULITE 2000 automated chemiluminescence-immunoassay system (Diagnostic Products Corporation, Los Angeles, CA).

Body mass index (BMI) was calculated by dividing weight in kilograms by the square of height in meters (kg/m^2^); patients with BMI ≥30 kg/m^2^ were considered obese according to the classification proposed by the World Health Organization [26]. Glucose intolerance was considered present when the 120 min value >140 mg/dL [27]. Insulin resistance was considered where 1/HOMA index <0.47 and QUICKI index ≤0.333 [8, 9]. Metabolic syndrome was characterized according to the National Cholesterol Education Program (NCEP) Adult Treatment Panel III (ATP III) and International Diabetes Federation (IDF) criteria [28–30]. TNF*α* values >8.1 pg/mL and IL-6 >5.9 pg/mL were considered abnormal.

The ophthalmologic evaluation protocol included measurement of corrected visual acuity, anterior segment slit-lamp biomicroscopy, fundus biomicroscopy with a Volk 78 D lens, measurement of intraocular pressure with a Goldmann tonometer, and retinography and optical coherence tomography of the posterior segment. For volunteers who were menstruating, the ophthalmologic evaluation was performed at the early follicular phase, in order to minimize possible interference of hormonal fluctuations of the menstrual cycle. All volunteers who were included in the study had visual acuity ≥20/40, intraocular pressure <21 mmHg, and spherical equivalent within ±5.00 D. After pharmacologic mydriasis with tropicamide eye drops, photographs of the fundus were taken with a Visucam NNM/FA fundus camera (Carl Zeiss Meditec, Dublin, California) and evaluated according to the Early Treatment Diabetic Retinopathy Study (ETDRS) grading system. A retinopathy severity score was assigned according to the modified Airlie House Classification System [31].

**Figure 1:**
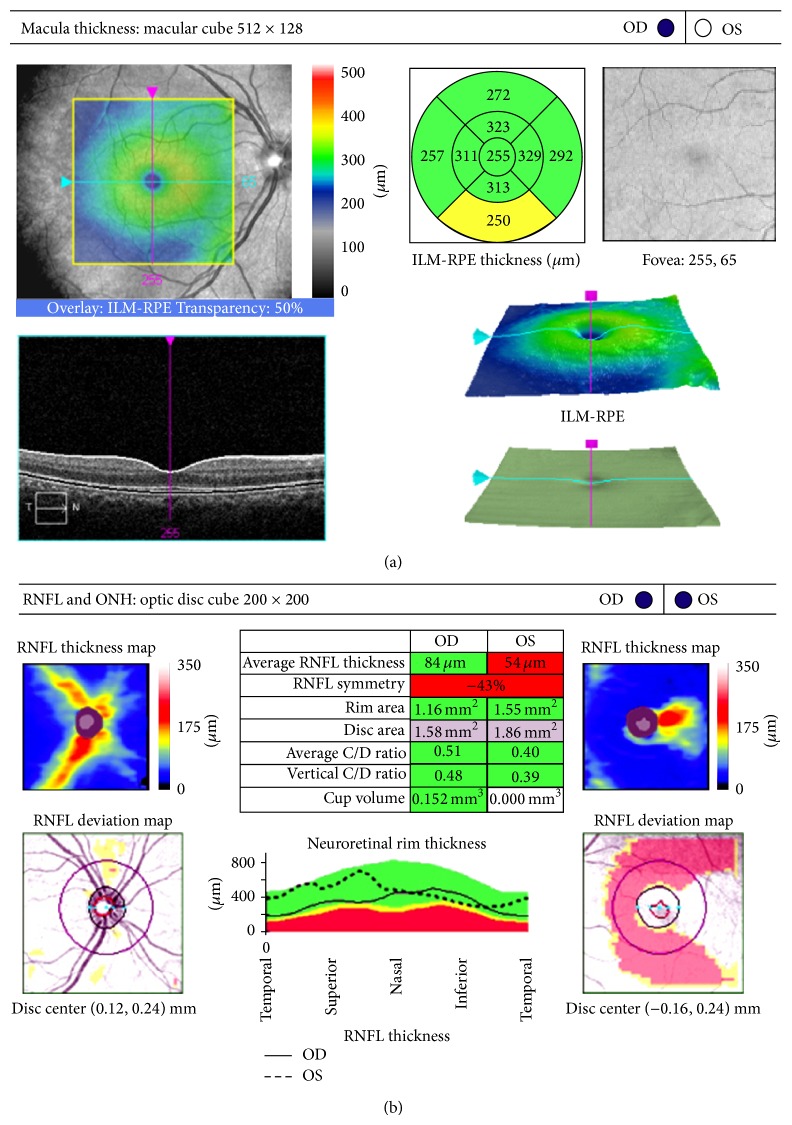
(a) “Macular Cube 512 × 128” software, measuring total macular thickness, producing a map with 09 subfields of sectoral thicknesses in three concentric circles, as defined by ETDRS. (b) “Optic Disc Cube 200 × 200” software for measuring the RNFL around the optic nerve, producing a map with 04 areas and optic disc parameters. The same software was used in the macular region to measure the thickness of the nerve fiber layer of the macula.

Cirrus spectral-domain HD-OCT (Carl Zeiss Meditec, Dublin, California) was performed to obtain measures of retinal thickness. For measurement of total macular thickness, “Macular Cube 512 × 128” software was used, where a macular scan is performed on a 6 mm × 6 mm area over 2.4 seconds, including 512 horizontal B-scans with 128 A-scans for each section and providing a map with 09 subfields of sectoral thicknesses in three concentric circles with diameters of 1, 3, and 6 mm, as defined by ETDRS (Figure 1(a)) [32, 33]. “Optic Disc Cube 200 × 200” software was used to measure the thickness of the RNFL in the optic nerve region, performing 200 horizontal B-scans with 200 A-scans for each section. The software automatically determines the center of the optic disc and measures nerve fiber thickness along a circular path with a radius of 1.73 mm in peripapillary diameter, providing a map of four areas (Figure 1(b)). The same “Optic Disc Cube 200 × 200” software was then used on the macular region and along a circular path with a radius of 1.73 mm in diameter. Centering on the fovea, we measured the thickness of the nerve fiber layer in the macular region (Figure 1(b)) [25]. Low-quality scans were excluded and defined as (A) signal strength <7; (B) decentration of the fovea or optic disc; and (C) image artifacts captured.

*Statistical Analysis.* The Kolmogorov-Smirnov test was used to evaluate data distribution. Student's *t*-test was used for quantitative variables with normal distribution, and the results are expressed as the mean ± standard deviation. The Mann-Whitney test was used for variables with nonparametric distribution that were expressed as median (25th–75th percentiles). The Chi-square and Fisher exact tests were adopted for qualitative variables and results are expressed as proportions. Size reduction was applied to the variables total macular thickness (09 variables) and RNFL around the optic nerve (04 variables) and the macula (04 variables) using Principal Component Analysis for each model. Association of principal components (PC1 and PC2) with clinical conditions was tested using a multivariate analysis of variance (MANOVA) model. A *P* value < 0.05 was considered statistically significant in all procedures.

## 3. Results

Table 1 shows a comparison of clinical, anthropometric, biochemical, hormonal, and inflammatory characteristics between the PCOS and control group volunteers. The prevalence rates of metabolic syndrome were 31.8% in patients with PCOS and 21.2% for control group volunteers, according to NCEP criteria (*P* = 0.301), and 31.8% and 24.2% in PCOS patients and controls, respectively, according to IDF criteria (*P* = 0.466). The prevalence rates of glucose intolerance were 20.9% in the PCOS group and 10.7% in the control group (*P* = 0.215). The prevalence rates of insulin resistance were 33.3% in the PCOS group and 9.1% in the control group (*P* = 0.01). Obesity was more frequent in PCOS group than in the control group (53.3% and 19.1%, resp.; *P* = 0.001). A significantly higher rate of women with altered inflammatory parameters was also observed in the PCOS group than in the control group (55.9% and 15.2%, resp.; *P* = 0.001).

From 45 eyes of the volunteers in the PCOS group, 42 were selected (3 eyes were excluded, 2 due to refractive errors > or < than 5.00 D, and 1 due to signal strength <7 when performing OCT). Likewise, from 47 eyes of the volunteers in the control group, 40 were selected (7 eyes were excluded, 4 due to refractive errors > or < than 5.00 D, and 3 due to signal strength <7 when performing OCT).

Tables 2 and 3 present a comparison between groups for HD-OCT results.  Table 4 shows a comparison of total macular thickness between the PCOS and control groups after metabolic stratification. The means of the 9 total macular thickness subfields were not significantly different between the PCOS and control groups. However, after stratification for the considered metabolic conditions, significant differences were observed in the areas temporal inner macula (TIM), inferior inner macula (IIM), nasal inner macula (NIM), and nasal outer macula (NOM), according to the presence of insulin resistance (Table 4). It is worth mentioning that, in the absence of insulin resistance, there were no statistically significant differences among the means of the measurements of the total macular thickness between the studied groups.

The mean value of the superior RNFL in the optic nerve region was significantly thicker in the PCOS group than in the control group. Additionally, the mean value of the cup volume (optic disc parameter) was significantly lower in the PCOS group than in the control group (Table 3).

The MANOVA model was used for principal components of retinal thickness and clinical conditions variables. Both the presence of obesity associated with insulin resistance (*P* = 0.037) and glucose intolerance (*P* = 0.001) were associated with significant increase in the PC1 mean score, relative to macular thickness. Still regarding total macular thickness, with the presence of metabolic syndrome there was a significant increase in the PC2 average score (*P* < 0.0001). Furthermore, there was a significant interaction between obesity and inflammation in a decreasing mean PC2 score relative to macular RNFL thickness (*P* = 0.034).

## 4. Discussion

The aim of this study was to evaluate changes in total macular thickness and in the RNFL in the macula and around the optic disc in women with PCOS in comparison with healthy ovulatory patients. This was achieved using HD-OCT, and results were stratified according to the presence of metabolic syndrome, inflammation, obesity, glucose intolerance, and/or insulin resistance. Our study is the first to analyze HD-OCT data for women with PCOS in association with metabolic changes. The results demonstrated that there was increased total macular thickness in patients with PCOS in the presence of insulin resistance compared to the control group. As for RNFL, there was increased thickness in the region around the optic nerve and decreased thickness in the macula, suggesting that retinal changes in PCOS differ according to eye region.

The endocrine, inflammatory, and metabolic changes associated with PCOS have consequences for vision, including neurovascular metabolic changes in the retina. Some studies have revealed fundoscopic alterations of the retina, such as retinal hemorrhages and exudates, in the absence of diabetes and diabetic retinopathy [34–36], and their association with inflammatory markers, metabolic syndrome, and other cardiovascular risk factors. The advent of OCT has brought about increased interest in the study of retinal microstructure in research focusing on early and preclinical diagnosis, as OCT is characterized by high resolution, accuracy, and reproducibility.

Metabolic stratification revealed increased total macular thickness in the TIM, IIM, NIM, and NOM subfields (insulin resistance). We speculate that this change is initially associated with hypertrophy and, subsequently, with intra- and extracellular edema of Müller cells. Both medical conditions can be recognized as early stages of retinopathy, even in asymptomatic patients with no visible changes on ophthalmoscopy and fluorescein angiography [22, 25, 37]. As confirmed by electrophysiological and psychophysical studies, changes in Müller cells precede microvascular events because these cells are susceptible to hyperglycemia and possibly to insulin resistance and inflammation [25, 37]; the result is an increase in the total thickness of the macular region. Our study also reveals a decrease in RNFL thickness in the macular region. This decrease occurs because abnormal Müller cells secondarily induce a progressive neuronal loss [37]. The inability to maintain adequate osmotic equilibrium between the intra- and extracellular milieu leads to caspase 3 activation [38], followed by apoptosis of retinal ganglion cells and astrocytes, and consequently axonal loss [25, 37].

Another finding in our study was related to RNFL thickness around the optic nerve, in which we observed an increase in patients with PCOS compared with the control group. Studies suggest that women with PCOS have elevated levels of NGF (nerve growth factor), which is required for ovarian follicular growth, and that this increase is induced by hyperandrogenism and elevated plasma LH concentrations [39]. Furthermore, testosterone is cited as having a neuroprotective action by crossing the blood-brain barrier and affecting nerve cells (neurons and glia) in their recovery or regeneration [40, 41]. Experiments with rats further suggest that testosterone is associated with an increase in the size of neuronal perikarya and their processes and with synaptogenesis [40]. Perrin et al. demonstrated that the growth of white matter exhibited obvious sexual dimorphism: the volume of white matter increased sharply in males compared with females, with evidence supporting the action of testosterone on myelination, affecting the axonal caliber and thickness of the myelin sheath [42]. Recently published research demonstrates a possible association between increased RNFL thickness around the optic nerve and PCOS [19]. Furthermore, we analyzed “optic disc parameter” variables that have not been described in the literature for these groups. The variables showed significant differences, corroborating the possible “neuroprotective” effect of testosterone.

## 5. Conclusion

So, is PCOS an aggressive or protective factor for the retina? One can imagine that different regions of the retina respond differently to the neuroprotective action of testosterone and to the neurodegenerative action of metabolic changes. In summary, our results suggest that PCOS may represent a protective factor in the RNFL around the optic nerve, possibly through the action of testosterone. Conversely, when associated with metabolic abnormalities, PCOS may represent an aggressive factor in the macular region (macular RNFL and total macular thickness). Prospective studies involving women with PCOS are needed to further elucidate this neuroprotection/neurodegeneration interface in relation to the sensorineural layer of the eye. Such studies may or may not lead to the use of this layer as an indicator of protection/compromise of the eye, which in turn would facilitate the establishment of preventive clinical strategies for women with PCOS.

## Figures and Tables

**Table 1:** Comparison of clinical, anthropometric, biochemical, hormonal, and inflammatory characteristics between PCOS and control groups.

	PCOS group	Control group	*P*
Age (years)	26.6 ± 4.7	26.8 ± 4.2	*0.787* ^1^
Body mass index (Kg/m^2^)	30.6 ± 6.0	27.2 ± 4.0	*0.002* ^1^
Waist circumference—WC (cm)	93.1 ± 15.0	87.7 ± 11.0	*0.049* ^1^
SBP—systolic blood pressure (mmHg)	115 (107.8–123)	110 (110–120)	*0.366* ^2^
1/HOMA	0.7 (0.3–1.4)	1.1 (0.7–1.5)	*0.031* ^2^
QUICKI	0.3591 (0.3-0.4)	0.3872 (0.3-0.4)	*0.031* ^2^
Total cholesterol (mg/dL)	154.9 ± 28.0	149.7 ± 25.6	*0.407* ^1^
HDL-cholesterol (mg/dL)	39.3 ± 10.5	43.4 ± 8.6	*0.077* ^1^
LDL-cholesterol (mg/dL)	95.6 ± 23.4	91.2 ± 26.2	*0.443* ^1^
Triglycerides (mg/dL)	113.2 ± 51.1	74.9 ± 30.8	*<0.0001* ^1^
Fasting glucose (mg/dL)	71.2 ± 15.9	74.1 ± 8.3	*0.292* ^1^
120 m glucose (mg/dL)	104.9 ± 25.4	100.6 ± 29.9	*0.525* ^1^
Urea (mg/dL)	24.5 ± 7.9	22.6 ± 4.6	*0.192* ^1^
Aspartate aminotransferase (U/L)	21.9 ± 7.3	16.4 ± 4.6	*0.001* ^1^
Alanine aminotransferase (U/L)	25.9 ± 11.8	17.4 ± 8.6	*0.002* ^1^
Total testosterone (ng/dL)	138.1 ± 95.9	109.5 ± 27.9	*0.049* ^1^
Luteinizing hormone—LH (mlU/mL)	5.7 ± 4.2	3.2 ± 4.6	*0.017* ^1^
Follicle-stimulating hormone—FSH (mlU/mL)	4.1 ± 2.2	2.7 ± 2.3	*0.011* ^1^
Fasting insulin (mIU/mL)	12.8 ± 11.5	6.6 ± 4.9	*<0.002* ^1^
Tumor necrosis factor—TNF-*α* (pg/mL)	7.1 ± 3.9	6.0 ± 1.4	*0.0129* ^1^
C-reactive protein (mg/L)	316.5 ± 214.7	44.9 ± 23.1	*<0.0001* ^1^
Interleukin-6 (pg/mL)	6.2 ± 2.2	3.7 ± 1.2	*<0.0001* ^1^

^1^Student's *t*-test, independent samples; ^2^Mann-Whitney test; PCOS: polycystic ovary syndrome; 1/HOMA: 1/homeostasis model assessment index; QUICKI: quantitative insulin sensitivity check index.

**Table 2:** Total macular thickness and RNFL in the macular region using HD-OCT: comparison between PCOS and control groups.

	PCOS group	Control group	*P*
Total macular thickness (*μ*m)			
Central subfield (CSF)	242.9 ± 19.7	241.2 ± 19.8	*0.701* ^1^
Superior inner macula (SIM)	320.0 ± 15.0	323.8 ± 14.1	*0.239* ^1^
Temporal inner macula (TIM)	305.5 ± 12.3	308.6 ± 14.2	*0.297* ^1^
Inferior inner macula (IIM)	316.3 ± 14.5	317.0 ± 21.4	*0.865* ^1^
Nasal inner macula (NIM)	322.5 ± 15.4	322.8 ± 15.5	*0.941* ^1^
Superior outer macula (SOM)	282.6 ± 15.0	285.6 ± 14.6	*0.358* ^1^
Temporal outer macula (TOM)	260.7 ± 12.6	263.5 ± 13.6	*0.349* ^1^
Inferior outer macula (IOM)	268.9 ± 14.8	268.8 ± 15.0	*0.980* ^1^
Nasal outer macula (NOM)	300.7 ± 14.1	299.4 ± 19.6	*0.737* ^1^
Retinal nerve fiber layer (macula, *μ*m)			
Average macular RNFL	38.6 ± 4.8	41.0 ± 9.5	*0.168* ^1^
Superior macular	33.9 ± 5.2	35.2 ± 7.3	*0.334* ^1^
Temporal macular	52.5 ± 15.0	54.2 ± 16.1	*0.636* ^1^
Inferior macular	33.8 ± 7.2	35.6 ± 7.0	*0.258* ^1^
Nasal macular	35.0 ± 11.3	35.2 ± 8.0	*0.945* ^1^

^1^Student's *t*-test, independent samples.

**Table 3:** The optic disc parameters and RNFL thickness around the optic nerve using HD-OCT: comparison between PCOS and control groups.

	PCOS group	Control group	*P*
Optic disc parameters			
Cup volume (mm^3^)	0.1 ± 0.1	0.2 ± 0.2	*0.001* ^1^
Average C/D ratio	0.40 ± 0.1	0.5 ± 0.2	*0.071* ^1^
Average vertical C/D ratio	0.4 ± 0.1	0.5 ± 0.2	*0.100* ^1^
Retinal nerve fiber layer (optic nerve, *μ*m)			
Average RNFL	101.2 ± 11.2	98.0 ± 9.0	*0.162* ^1^
Superior	128.0 ± 18.0	120.0 ± 15.1	*0.036* ^1^
Temporal	66.3 ± 9.7	65.4 ± 8.7	*0.652* ^1^
Inferior	135.0 ± 17.5	130.6 ± 15.0	*0.222* ^1^
Nasal	75.8 ± 14.5	77.6 ± 9.9	*0.512* ^1^

^1^Student's *t*-test, independent samples.

**Table 4:** Total macular thickness using HD-OCT: comparison between PCOS and control groups after metabolic stratification.

	PCOS group + insulin resistance	Control group + insulin resistance	*P* ^1^
Total macular thickness (μm)			
Temporal inner macula (TIM)	304.5 ± 7.5	292.7 ± 11.0	0.035
Inferior inner macula (IIM)	316.3 ± 12.3	288.0 ± 33.0	0.017
Nasal inner macula (NIM)	324.1 ± 11.9	302.0 ± 15.6	0.010
Nasal outer macula (NOM)	301.3 ± 13.2	283.0 ± 4.6	0.035

^1^Student's *t*-test, independent samples.
